# MD-2 Determinants of Nickel and Cobalt-Mediated Activation of Human TLR4

**DOI:** 10.1371/journal.pone.0120583

**Published:** 2015-03-24

**Authors:** Alja Oblak, Jelka Pohar, Roman Jerala

**Affiliations:** 1 Department of Biotechnology, National institute of Chemistry, Ljubljana, Slovenia; 2 Centre of Excellence EN-FIST, Ljubljana, Slovenia; INSERM, FRANCE

## Abstract

Recent findings unexpectedly revealed that human TLR4 can be directly activated by nickel ions. This activation is due to the coordination of nickel by a cluster of histidine residues on the ectodomain of human TLR4, which is absent in most other species. We aimed to elucidate the role of MD-2 in the molecular mechanism of TLR4/MD-2 activation by nickel, as nickel binding site on TLR4 is remote from MD-2, which directly binds the endotoxin as the main pathological activator of TLR4. We identified MD-2 and TLR4 mutants which abolished TLR4/MD-2 receptor activation by endotoxin but could nevertheless be significantly activated by nickel, which acts in synergy with LPS. Human TLR4/MD-2 was also activated by cobalt ions, while copper and cadmium were toxic in the tested concentration range. Activation of TLR4 by cobalt required MD-2 and was abolished by human TLR4 mutations of histidine residues at positions 456 and 458. We demonstrated that activation of TLR4 by nickel and cobalt ions can trigger both the MyD88-dependent and the –independent pathway. Based on our results we propose that predominantly hydrophobic interactions between MD-2 and TLR4 contribute to the stabilization of the TLR4/MD-2/metal ion complex in a conformation that enables activation.

## Introduction

Allergic contact dermatitis (ACD) is one of the most common occupational skin diseases. In the first asymptomatic stage of the disease, termed sensitization phase, dendritic cells present contact allergens (*i*.*e*. haptens) to T lymphocytes, which are the effector cells responsible for the typical skin rash in the second, elicitation phase that follows the re-exposure to the allergen [1]. Over 3000 contact allergens are known, with nickel being one of the most common ones. The prevalence of nickel hypersensitivity is 19,7–24,5% across Europe [2]. With such high prevalence measures have been taken to reduce the content of nickel in different alloys. ACD still remains a major problem since a range of everyday objects contains traces of allergenic metals, *e*.*g*. some stainless steels, colored pottery and even medical implants contain cobalt. Although cobalt is a trace element found in the body as the cofactor for vitamin B12, higher concentrations are highly toxic and can cause major health problems, including ACD [3].

The immune response underlying all allergic reactions is a result of a collective action of the adaptive and innate immune system. TLR4/MD-2 receptor complex is one of the pattern recognition receptors that represent the first line of innate immune defense against pathogenic microorganisms [4]. TLR4/MD-2 recognizes endotoxin (*i*.*e*. LPS, lipopolysaccharide), a major constituent of the Gram-negative bacterial cell wall, which binds into a hydrophobic pocket formed by MD-2 [5]. The molecular mechanism of TLR4/MD-2 activation has been elucidated based on the crystal structure of the activating complex [6] and mutational studies [7]; they have revealed that endotoxin acyl chains contribute to the formation of a solvent-exposed hydrophobic surface that mediates interactions between MD-2 and the opposing TLR4, which drives receptor heterodimerisation and subsequent signal triggering that leads to immune cell activation.

Several studies have proposed a range of alternative endogenous TLR4/MD-2 ligands, but doubts remain concerning the presence of LPS-like contaminants [8]. Until recently only endotoxin (and taxol in the murine receptor complex [9]) was being considered as a *bona fide* TLR4/MD-2 ligand. Schmidt *et al*. made an intriguing discovery that nickel can specifically activate the human TLR4/MD-2 receptor complex [10]. They identified three histidine residues in the TLR4 ectodomain; two of them are present in human and primates, but are not conserved in most other species. These histidines were proposed to coordinate the binding of nickel ions between two TLR4 ectodomains, which would trigger TLR4 dimerization and activation. Indeed, when these histidines were mutated, nickel could no longer induce TLR4 activation. Interestingly, TLR4 activation did not take place in the absence of MD-2 even though the nickel binding site on the TLR4 ectodomain is distinct from MD-2 or from the TLR4/MD-2 interaction interface and no direct contact between nickel and MD-2 was implied [10]. Surprisingly, TLR4 dimerization (but not activation) is independent of MD-2 [11]. Moreover, cobalt ions can also mediate TLR4 dimerization independently of MD-2, but still require MD-2 for cellular activation [11].

In our study we addressed the question of the role of MD-2 in cell activation by metal ions. We provide evidence that the nickel binding site is completely independent of the endotoxin binding site. We propose a molecular model in which MD-2 contributes to the interaction with a hydrophobic patch on the surface of TLR4, which helps stabilize the TLR4/MD-2/Ni^2+^ complex in a proper conformation that enables cellular activation. Moreover, we provide evidence that both nickel and cobalt follow the same mechanism of activation of the human TLR4/MD-2 receptor complex, while copper and cadmium ions are unable to trigger TLR4/MD-2-mediated cellular activation.

## Materials and Methods

### Reagents

Expression plasmids containing sequences of human TLR4 and MD-2 as well as the pELAM-1 firefly luciferase plasmid were a gift from Dr. C. Kirschning (Technical University of Munich, Germany). Plasmid encoding the firefly luciferase under IFN-β promoter was a gift from Dr. J. Hiscott [12] [Dep. of microbiology and medicine, McGill University, Montreal, QC, Canada]. Plasmid encoding the firefly luciferase under IP-10 promoter was a gift from Dr. R. Ransohoff [13] (Dep. of Neurosciences, Lerner Research Institute, Cleveland Clinic Foundation, Cleveland, OH, USA). Expression plasmid containing the sequence of mouse TLR4 was purchased from InvivoGen (CA, USA). Expression plasmid for mouse MD-2 was a gift from Dr. Y. Nagai (University of Tokyo, Japan). The Renilla luciferase phRL-TK plasmid was purchased from Promega (WI, USA). The nucleotide sequence encoding human TLR4 was cloned into pUNO vector with a C-terminal HA tag. Transfection reagent JetPEI was purchased from Polyplus-Transfection (France) and was used according to the manufacturer’s instructions. Endotoxin (LPS) was from *Escherichia coli* 055:B5 (Sigma). Synthetic tetraacylated lipid IVa (compound 406) was purchased from Peptide Inc. (Japan). Metal ions (nickel II chloride hexahydrate, cobalt II chloride, copper II chloride dihydrate and cadmium chloride) were purchased from Sigma.

### Cell cultures

The human embryonic kidney (HEK) 293 cells were provided by Dr. J. Chow (Eisai Research Institute, Andover, USA). HEK293 cells were used in cell activation assays with TLR4 mutants. Flp-In T-REx cells (Invitrogen), derived from HEK293 cells, and the Flp-In system were purchased from Invitrogen (CA, USA). T-REx cell lines stably expressing human or mouse TLR4 (named HEK/TLR4) were made using the Flp-In system according to the manufacturer’s instructions. Briefly, human and mouse TLR4 nucleotide sequences were cloned from the pUNO-HA vector into pcDNA5/FRT expression vector, which was then transfected into Flp-In T-REx cells along with pOG44 expression plasmid for expression of the Flp recombinase. After homologous recombination between the FRT sites in the T-REx cell genome and the pcDNA5/FRT vector, stable cells were selected for hygromycin resistance. Both HEK293 and T-REx cells (HEK293/TLR4) were grown in DMEM supplemented with 10% FBS. Primary human umbilical vein endothelial cells (HUVEC-dLy) were purchased from Lonza. They were cultivated in microvascular endothelial cell growth medium-2 (EGM2-MV BulletKit; Lonza) according to the supplier’s recommendations. THP-1 cells were purchased from Sigma-Aldrich and were cultured in RPMI medium supplemented with 10% FBS.

### Site-directed mutagenesis

All MD-2 mutants were made using QuikChange site-directed mutagenesis kit (Stratagene, USA) according to the manufacturer’s instructions. All plasmids were sequenced to confirm the mutation.

### Cell activation assay—NF-κB-luciferase reporter assay

The TLR4/MD-2 activation was evaluated by monitoring the expression of a firefly luciferase under the control of downstream inducible promoters (NF-κB-, IFNβ- or IP10-dependent promoters). HEK293 or HEK293/TLR4 cells were seeded in 96-well Costar plates (Corning, NY, USA) at 3,5∙10^4^ cells/well and incubated overnight in a humidified atmosphere (5% CO2) at 37°C. The next day, when cells were 60–80% confluent, they were co-transfected with pEF-BOS-MD-2 (10 ng), NF-κB-dependent luciferase (50 ng) and constitutive Renilla (10 ng) reporter plasmids and (in the case of HEK293 cells) pUNO-TLR4 plasmid (1 ng) using JetPEI transfection reagent. For evaluation of the MyD88-independent pathway activation, plasmids encoding the IFNβ- or IP10-dependent firefly luciferase were transfected in place of the NF-κB-dependent luciferase. Cells were stimulated the next day for 6 hours (unless indicated differently in figure legends) with the indicated concentration of ligands. Cells were then lysed in 1x reporter assay lysis buffer (Promega, USA) and analyzed for reporter gene activities using a dual-luciferase reporter assay system. Relative luciferase units (RLU) were calculated by normalizing each sample’s firefly luciferase activity for constitutive Renilla activity measured within the same sample. When plotting the data the value of the control (wild type TLR4/MD-2 stimulated with LPS) was normalized to 100 and other values were adjusted accordingly. Experiments were independently performed at least three times with similar results, each time in at least three replicates. Figures show results from a representative experiment with standard deviation. The p values were calculated using Student one-tailed t-test.

### ELISA

Human IL-8 concentrations were determined in the supernatants of HUVEC cells. Cells were seeded at 7∙10^4^ cells/well in 100 μl on a 96-well plate. They were stimulated the next day with 100 ng/ml endotoxin or 1 mM nickel or cobalt ions for 6 or 16 hours. Cell culture medium was used as a control/mock. The supernatants were then tested using Platinum hIL-8 ELISA kit (eBioscience). For measuring the cytokines in the supernatants of THP-1 cells (undifferentiated monocytes), the cells were seeded in a 24-well plate (3∙10^5^ cells/well in 0,5 ml of medium) and stimulated with LPS or metal ions at concentrations indicated in the figures (or were mock stimulated using cell culture medium for the negative control). After 16 hours, the supernatants were harvested and the amount of human IL-6, IL-8 and TNFα was determined using ReadySetGo ELISA kits (eBioscience). The p values were calculated using Student one-tailed t-test.

### SDS-PAGE and western analysis

The phosphorylation of the IκBα protein after LPS or metal-ion stimulation was determined using the THP-1 macrophages. THP-1 cells were seeded in a 24-well plate (3∙105 cells/well in 0,5 ml of medium) and were differentiated into macrophages with 5 ng/ml PMA (phorbol 12-myristate 13-acetate) for three days (prior to stimulation) and were then stimulated with LPS (100 ng/ml) or metal ions (2 mM) for the indicated time. The cells were then resuspended in a lysis buffer (Tris 50 mM, NaCl 150 mM, Triton X-100 1%, SDS 0,1%, DOC 0,5% with Phosphatase inhibitor cocktail (Calbiochem) and Complete protease inhibitor cocktail (Roche Applied Science)), kept on ice for 15 minutes and centrifuged at 4°C. The supernatants were harvested and the total protein amount was quantified using the BCA protein assay (Sigma). Cell lysates were incubated at 95°C for 3 min with sample buffer (SDS with 2-mercaptoethanol) and loaded onto a 12% SDS-PAGE gel (30 μg of total proteins/well) along with a protein ladder (Thermo Scientific). After electrophoresis the proteins were transferred onto a nitrocellulose membrane using iBlot Gel Transfer Device (Life Technologies). The membrane was blocked with 5% non-fat dry milk in TBS and then stained with anti-phospho primary antibodies (phospho-IκBα (Ser32/36) (5A5) mouse mAb diluted 1:1000—Cell Signaling) and anti-tubulin (α/β-Tubulin rabbit pAb 1:5500—Cell Signaling) antibodies. The membrane was then stained with goat anti-mouse IgG-HRP (diluted 1:3000—Santa Cruz) and Goat Anti-Rabbit IgG-HRP (diluted 1:4500—Abcam). The blots were developed using SuperSignal West Femto Chemiluminescent Substrate (Pierce). Membranes were recorded with G:BOX Chemi using GeneSnap software (Syngene).

## Results

### The activation of hTLR4/MD-2 by nickel and cobalt is not antagonized by the LPS antagonist lipid IVa

**Fig 1:**
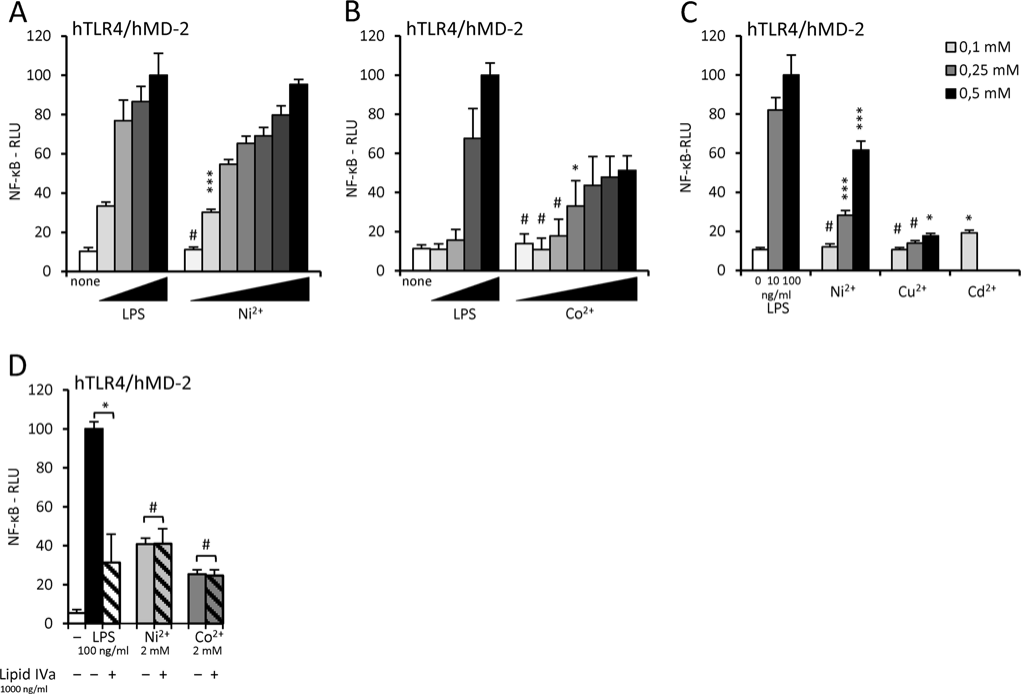
Nickel and cobalt trigger NF-κB activation, which is not inhibited by lipid IVa. (A, B) Dose-dependent activation of human TLR4/MD-2 by nickel and cobalt. HEK293/hTLR4 cells were transfected with plasmid encoding hMD-2 and luciferase reporter plasmids. Cells were stimulated with LPS (0, 1, 5, 10, 100 ng/ml) and nickel (A) or cobalt (B) (0.10, 0.25, 0.50, 0.75, 1.0, 2.0, 4.0 mM) for 6 hours, lysed and tested for luciferase activity. (C) Copper and cadmium do not activate human TLR4/MD-2 receptor complex at low, non-toxic concentrations. (D) Tetraacylated lipid IVa (compound 406) does not inhibit human TLR4 activation by nickel or cobalt ions. #p≥0,01 (not significant); *p<0,01; ***p<0,0001 (t-test, compared to the unstimulated control unless indicated otherwise by brackets).

The intriguing results from Schmidt *et al*. [10] encouraged us to further elucidate the mechanism of TLR4/MD-2 receptor complex activation by nickel with an emphasis on the riddle why MD-2 is required for this type of TLR4 activation. First we wanted to confirm that nickel activates human TLR4 in our cellular system using cells stably expressing human TLR4. We observed a distinct dose-dependent activation when stimulating cells with nickel for as little as 6 hours (Fig. 1A). This NF-κB-dependent response was observed at later time points as well (data not shown), but due to the toxicity of nickel and consequently poorer cell viability activation at later time points could not be reliably quantified. However, with nickel concentrations used in experiments on HEK293/TLR4 cells, no decrease in cell viability was observed for stimulations of up to 10 hours.

Since histidine residues can also coordinate other ions besides nickel, we wondered whether any other metal ions of similar physical properties could activate human TLR4/MD-2 receptor complex in a similar manner. We first tested cobalt ions and observed a potent dose-dependent response in NF-κB activation (Fig. 1B). Further we tested copper and cadmium ions, both of which have the same charge and similar radii as nickel. We showed that neither copper nor cadmium is able to activate TLR4/MD-2 complex (Fig. 1C). We tested a range of concentrations of both ions (data not shown) but observed a strong toxic effect of copper (concentrations higher than 0,5 mM) and especially cadmium even at concentrations as low as 0,1 mM and stimulation times of under 300 minutes (data not shown). Since lower cell viability can affect the expression of the Renilla luciferase used for normalization, slight increase in the relative luciferase values can be attributed to cell toxicity. At sufficiently low concentrations where no toxicity was observed after 6 hour stimulation (*i*.*e*. under 0,1 mM) no activation was detected. Our results, which are in agreement with others [11,14], therefor confirm nickel and cobalt as potent cell activators.

We further tested whether lipid IVa, a tetraacylated endotoxin precursor that antagonizes activation of human TLR4/MD-2 by hexaacylated endotoxin [15], affects activation by nickel ions. Since the nickel and cobalt binding site is proposed to be distinct from the endotoxin binding site [10,11], any effect of lipid IVa on metal ion-induced activation would either question this proposed model of activation or implicate a lipid IVa-mediated repulsion in the TLR4/MD-2 complex that could not be overcome by the coordination of metal ions between the TLR4 ectodomains. While lipid IVa significantly inhibited activation by endotoxin in cells expressing human TLR4/MD-2, it had absolutely no effect on activation by nickel or cobalt (Fig. 1D). This confirms that the endotoxin and metal ion binding sites are distinct and moreover, it excludes the possibility of endotoxin contamination of our metal ion preparations or induction of any endogenous TLR4 activators that could bind to MD-2.

**Fig 2:**
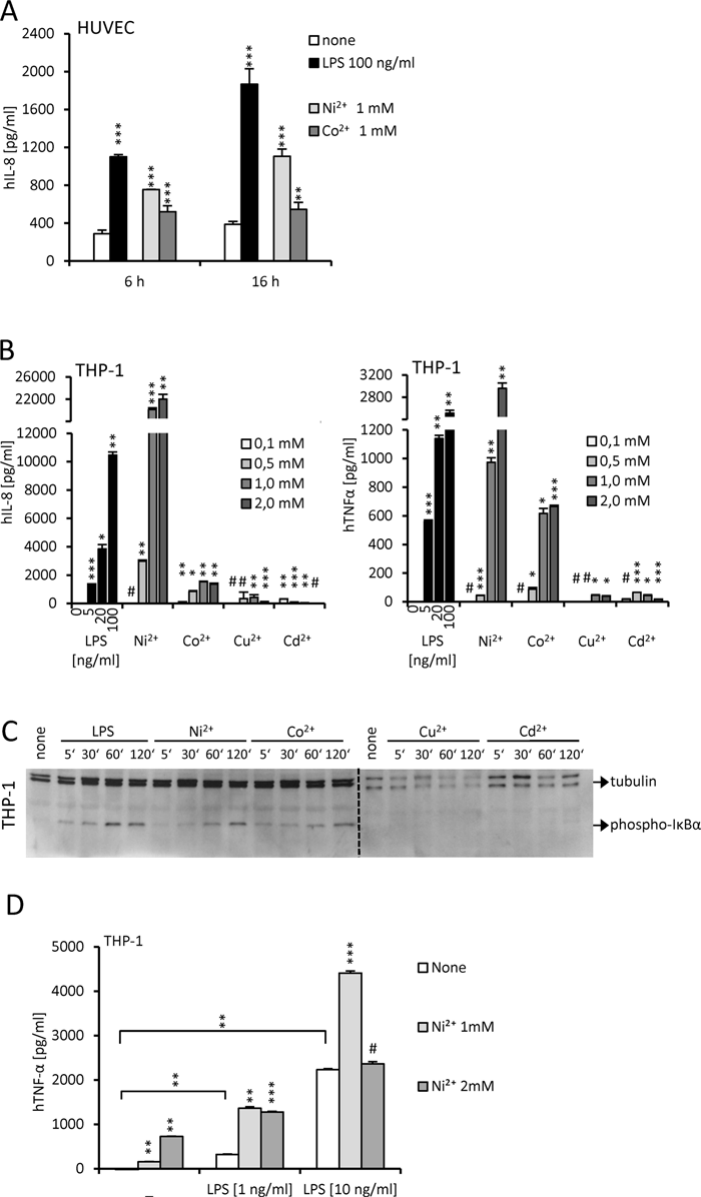
Nickel and cobalt trigger cytokine production and phosphorylation of intracellular signaling proteins. (A) Nickel and cobalt stimulation elicits hIL-8 production. HUVEC cells were stimulated with LPS, nickel or cobalt for either 6 or 16 hours. hIL-8 concentration was measured in cell culture supernatants by ELISA. (B) THP-1 cells were stimulated with LPS or metal ions for 16 hours. Cytokine concentration was measured in cell culture supernatants by ELISA. (C) THP-1 cells were stimulated and the phosphorylation of the inhibitor of kappaB-alpha was detected with western blot. #p≥0,01 (not significant); *p<0,01; **p<0,001; ***p<0,0001 (t-test, compared to the unstimulated control). (D) THP-1 cells were stimulated with LPS alone or simultaneously with nickel for 6h. hTNF-α concentration was measured in cell culture supernatants by ELISA. #p≥0,01 (not significant); *p<0,01; **p<0,001; ***p<0,0001 (t-test, compared to the unstimulated control (as indicated by brackets) or compared to the cells not treated with nickel).

Nickel/cobalt-induced cell activation was detected not only on the transcription, but also on the cytokine production level with increase in IL-8 production by HUVEC (Fig. 2A) and THP-1 (Fig. 2B) cells. The IL-8 production by HUVEC cells was evident after just 6 hours of stimulation. Although HUVEC cells (as well as THP-1 cells) appeared to be less sensitive to metal ion toxicity (compared to HEK293 cells) and retained unimpaired viability, IL-8 production by HUVEC cells was just slightly increased after 16 hours of stimulation in comparison to 6 hour stimulation. IL-8 production by THP-1 cells induced by cobalt was modest compared to the potent activation induced by nickel (Fig. 2B). Nonetheless, cobalt induced strong TNFα production. Cytokine production induced by copper and cadmium was minute, yet still significantly above the mock-stimulated control. To evaluate the activation of the intracellular signaling cascades triggered by metal ion stimulation, we monitored the phosphorylation of the inhibitor of kappa B alpha (Fig. 2C). Nickel and cobalt induced strong phosphorylation of IκBα. On the other hand, phosphorylation of IκBα was not induced by copper or cadmium ions. Since the infection by Gram-negative bacteria of persons exposed to the environmental sources of nickel might be relevant we investigated the activation of human THP-1 cells. Results augmented production of TNFα by stimulation with both LPS and Ni (Fig. 2D). The effect was synergistic at concentration of LPS of 1 ng/ml. At higher concentration of LPS (10 ng/ml) the synergistic effect was observed only at lower concentrations of nickel (1 mM). Cobalt however did not augment production, probably due to the toxicity (data not shown).

These results show that, in contrast to copper and cadmium, nickel and cobalt induce potent NF-κB-dependent cell activation. Nickel, but not cobalt, can act synergistically with LPS.

### TLR4 activation by metal ions is mediated by histidine residues of the TLR4ecd but requires MD-2

**Fig 3:**
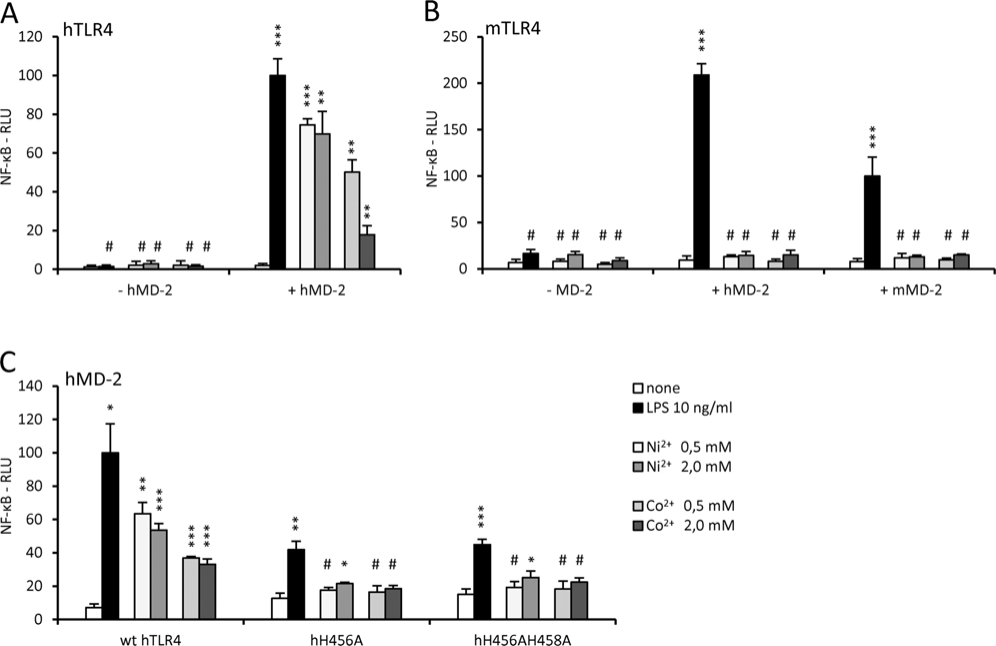
Nickel and cobalt activate HEK293 cells via human TLR4 and require MD-2 for activation. (A) HEK293/hTLR4 cells were transfected with the luciferase reporter plasmids with or without the plasmid encoding hMD-2. After stimulation, the luciferase activity was measured. (B) Mouse TLR4 with either mouse or human MD-2 does not support activation by nickel or cobalt. HEK293/mTLR4 cells were transfected with the luciferase reporter plasmids with or without plasmid encoding MD-2. (C) Mutation of histidines at positions 456 and 458 in hTLR4 nearly abolishes responsiveness to nickel and cobalt ions. HEK293 cells were transfected with luciferase reporter plasmids, plasmid encoding hMD-2 and plasmid encoding either wild type or mutant hTLR4. Luciferase activity was measured as indicated in Methods. #p≥0,01 (not significant); *p<0,01; **p<0,001; ***p<0,0001 (t-test, compared to the unstimulated control). The chart legend applies to all three panels.

In order to test whether metal ion activation of HEK293 cells is indeed dependent on human TLR4 and MD-2, we stimulated human TLR4-expressing cells with or without the co-expression of MD-2. No NF-kB-dependent response was detected in the absence of MD-2 (Fig. 3A), showing that MD-2 is an essential player not only in nickel but also in cobalt induced cell activation. Moreover we demonstrated that it is crucial that cells express human and not mouse TLR4, since neither of the tested metal ions could stimulate mTLR4-expressing cells regardless of the MD-2 species (Fig. 3B).

Nickel and cobalt ions are proposed to be coordinated by a cluster of non-conserved histidine residues, which are unique to human TLR4 [10,11]. Though also affecting endotoxin activation to a small degree, the H456A and the H456AH458A mutations in the human TLR4 ectodomain totally abolished activation by both nickel and cobalt (Fig. 3C).

### Stabilizing hydrophobic interactions between MD-2 and TLR4 are required for activation by metal ions

Since the binding site for both metal ions indeed seems to be completely distinct from the endotoxin binding site despite the apparent need for the presence of MD-2 in metal ion-induced activation, we then focused on the possible interactions between TLR4 and MD-2 that might contribute to the receptor complex stabilization. The dimerization interface between TLR4 and the opposing MD-2 that is established when TLR4/MD-2 receptor complex is activated by endotoxin includes hydrophobic interactions of an endotoxin acyl chain with specific amino acid side chains in both TLR4 and MD-2. Some of these hydrophobic amino acid residues are indispensable for activation by endotoxin, *e*.*g*. MD-2 residues valine 82 and leucine 87 and TLR4 residue phenylalanine 440 [7]. If the dimerization of TLR4, mediated by nickel or cobalt, proceeds through histidine residues, the residues mediating endotoxin-induced dimerization should have minimal effect on the activation by nickel.

**Fig 4:**
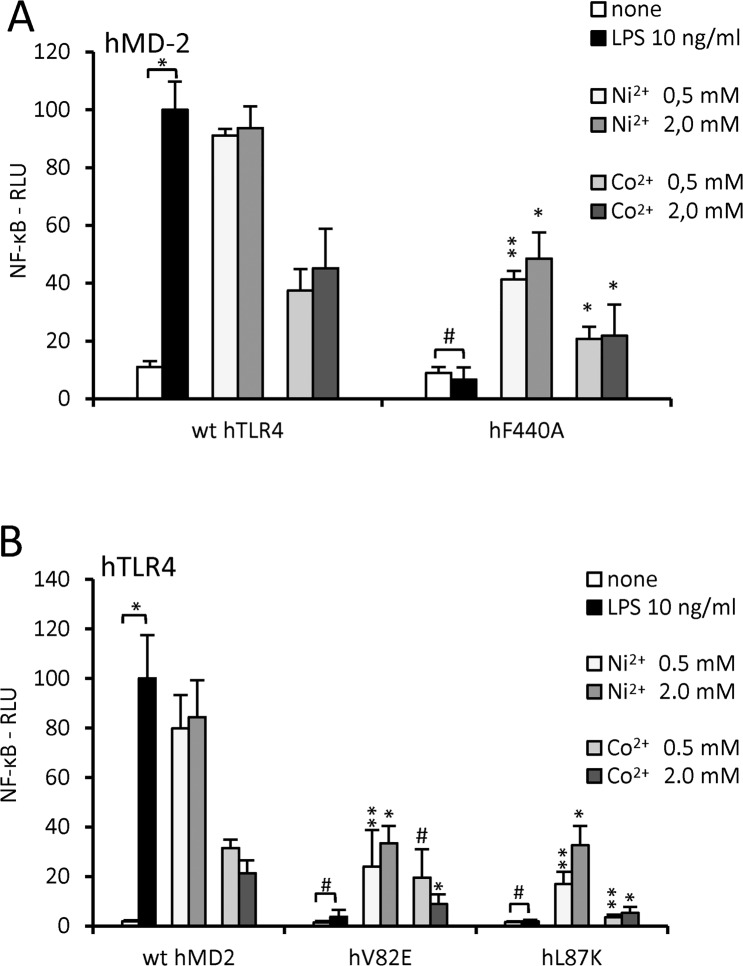
Activation of human TLR4 by metal ions requires different TLR4 and MD-2 residues than activation by endotoxin. (A) hTLR4 amino acid residues that are essential for activation by LPS are not needed for activation by nickel or cobalt. HEK293 cells were transfected with luciferase reporter plasmids, plasmid encoding hMD-2 and plasmid encoding either wild type hTLR4 or the hTLR4 mutant hF440A. (B) hMD-2 amino acid residues that are essential for activation by LPS are not needed for activation by nickel or cobalt. HEK293/hTLR4 cells were transfected with luciferase reporter plasmids and with plasmid encoding either wild type or mutant hMD-2. #p≥0,01 (not significant); *p<0,01; **p<0,001; (t-test, compared to the unstimulated control (as indicated by brackets) or compared to the wt hTLR4 with the corresponding treatment).

Mutation of a crucial TLR4 amino acid residue F440 completely abolished endotoxin signaling (Fig. 4A, [7]). Despite this fact the effect on the activation by nickel and cobalt was relatively small (Fig. 4A).

We then focused on MD-2 residues (positions 82 and 87) that contribute to the hydrophobic interactions that are indispensable for TLR4/MD-2 activation by endotoxin [6,7]. Replacing either V82 or L87 with a charged amino acid residue totally abolished the endotoxin-induced activation (Fig. 4B, [7]). The activation by nickel and cobalt was also affected, but to a lower extent (Fig. 4B). These results support the findings of Schmidt *et al*. [10] that proposed that the binding site for nickel ions in the TLR4/MD-2 complex is distant from the dimerization interface established when TLR4/MD-2 receptor complex is activated by endotoxin. Results in Fig. 4 further exclude the possibility of endotoxin contamination in our nickel preparation due to the fact that endotoxin could not activate cells when transfected with our TLR4 or charged MD-2 mutants. Nevertheless, a role of this hydrophobic amino acid-rich surface of both MD-2 and TLR4 is indicated, since we did observe lower NF-κB activation in response to metal ion stimulation of cells transfected with MD-2 or TLR4 mutants.

### Metal ions activate both MyD88-dependent and -independent pathways of the TLR4/MD-2 signaling

**Fig 5:**
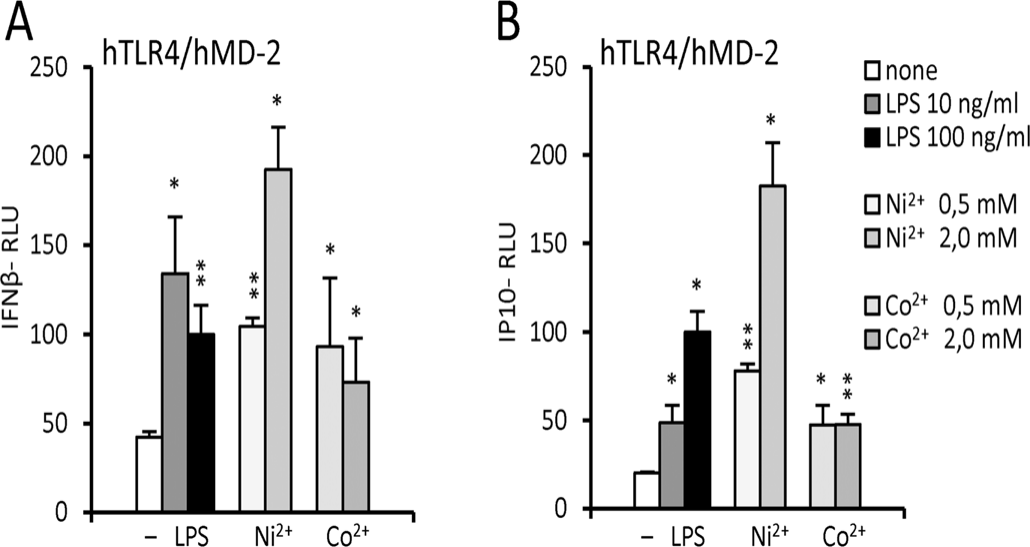
Nickel and cobalt activate MyD88-dependent and -independent pathways. HEK293/hTLR4 cells were transfected with luciferase reporter plasmids (constitutive Renilla-luciferase reporter and inducible) (A) IFNβ-responsive or (B) IP-10-responsive firefly-luciferase reporter) and with plasmid encoding wild type hMD-2. *p<0,01; **p<0,001; (t-test, compared to the unstimulated control). The chart legend applies to both panels.

Nickel was proposed to be a MyD88-specific TLR4 ligand due to the protonation of histidines at lower pH in the endosomes [16], which would prevent the coordination of nickel. We therefore tested whether nickel and cobalt are indeed incapable of activating the MyD88-independent TLR4 signaling pathway. We observed significant activation of both IFN-β- and IP-10-dependent promoters, indicative of the MyD88-independent pathway activation, upon stimulation by either nickel or cobalt (Fig. 5). These results suggest that nickel and cobalt are capable of activating both branches of the TLR4 signaling pathway.

## Discussion

Our study further elucidates the mechanism of metal-ion induced activation of the human TLR4/MD-2 complex, important for the nickel- and cobalt-induced allergic hypersensitivity, and sheds light on the requirement for MD-2 to be present in the activating complex. Our results are in agreement with the research by Schmidt *et al*. [10] who have demonstrated activation of human TLR4/MD-2 by nickel, thus identifying a totally new class of TLR agonists. Moreover, other contact hypersensitivity related transition metals such as cobalt and palladium have also recently been shown to activate TLR4 [11,14,17,18]. On the other hand, chromium, iron and zinc displayed no TLR4/MD-2 activating potential. In contrast to many other proposed alternative TLR4 ligands, where the possibility of endotoxin contamination could not be excluded [8], metal ion preparations used indeed seem to be totally endotoxin-free, as shown with limulus assays [10], polymyxin B [14] and in the present study with lipid IVa antagonism (Fig. 1D) and through the different effect of nickel/cobalt (vs. LPS) on the activation of the TLR4 and MD-2 mutants (Fig. 4).

Interestingly, the positioning of lipid IVa into the MD-2 binding pocket is markedly different in human and mouse MD-2 [19–21]. In human MD-2 [20], where lipid IVa acts as an antagonist of the activation by hexaacylated lipid A [15], the glucosamine backbone of lipid IVa is turned by approximately 180 degrees compared to its binding to the mouse MD-2 [19], where lipid IVa acts as an agonist. Ionic interactions between the phosphate groups of lipid IVa and specific amino acid residues of TLR4 and MD-2 have been proposed to be involved in lipid IVa species-specific recognition [22,23]. The proposed repulsion between lipid IVa and human TLR4 did not seem to interfere with activation of hTLR4/MD-2 by nickel or cobalt, since we did not observe any reduction of metal ion-induced activation when lipid IVa was present in the complex (Fig. 1D). We tried to expand the repertoire of the tested transition metals by performing experiments with copper and cadmium ions, but did not observe any TLR4/MD-2 activation when using low, non-toxic concentrations (Fig. 1C). Any apparent increase in the relative reporter activation was due to concomitant decrease in cell viability. Copper was also recently tested by Rachmawati *et al*. [14] who observed weak cell activation but were unable to attribute the response to TLR4 stimulation, since HEK293 cells without TLR4 or MD-2 expression surprisingly produced higher levels of IL-8 than the TLR4/MD-2 expressing cells did. We observed phosphorylation of the p38 MAP kinase (data not show), which also indicates the activation of stress-related pathways that may not be related to TLR4 activation. It is plausible that other transition metals of atomic radii and charge similar to nickel (like copper or cadmium) could trigger *in vitro* TLR4 ectodomain dimerization as they are efficiently chelated by imidazole [24], but could not be demonstrated due to high cell toxicity.

Since the metal ion binding site is clearly distinct from the endotoxin binding site, we next focused on the possible interactions between TLR4 and MD-2 that could contribute to complex stabilization and therefore activation. Specific hydrophobic amino acid residues in both TLR4 and MD-2 have been shown to be critical for activation by endotoxin by contributing to the formation of hydrophobic interactions that bind the activating TLR4/MD-2/endotoxin complex [7]. Mutations in these residues (TLR4_F440A, MD-2_V82E, MD-2_L87K), which disrupt their hydrophobic character, completely abolished activation by endotoxin. Activation by nickel and cobalt remained potent; nonetheless a reduced activation was observed (Fig. 4A,B). While these results confirm that metal ion and endotoxin binding sites are mutually independent, the lower activation by nickel and cobalt of charged mutants indicates that there is an interaction between MD-2 and TLR4 that contributes to metal ion-induced activation and is partially disturbed by these mutations. The crystal structure of the activating human TLR4/MD-2 complex shows hydrophobic interactions between the F440 of TLR4 and the F126 and I124 of MD-2 as well as interactions of both L87 and V82 of MD-2 with the F463 of TLR4 [6]. Although not sufficient to promote receptor complex autoactivation due to autoinhibitory characteristics of the TLR4 ectodomain [25], nickel or cobalt coordination between the histidines of the human TLR4 ectodomain appears to be sufficient to trigger receptor complex activation supported by the hydrophobic interactions between these amino acid residues (Fig. 4). Since the hydrophobic interactions between MD-2 and TLR4 are extensive, introducing single point mutations in the MD-2 or TLR4 ectodomain reflects in still significant but somewhat lower metal-induced TLR4 activation.

The coordination of metal ions between the histidines of the TLR4 ectodomain should in principle not be possible in acidic environments due to the protonation of histidine residues at pH lower than 6. Since the activation of the TRIF-dependent (MyD88-independent) pathway of TLR4 signaling occurs from acidic endosome vesicles [26–28], nickel was proposed to be an agonist that is exclusive to the MyD88-dependent pathway [16]. We tested this hypothesis by using IFNβ- and IP-10-responsive promoters in our reporter assays. Although cell activation with these reporters is markedly lower then with the NF-κB-dependent promoter even with endotoxin, we did detect significant activation of both promoters with nickel as well as cobalt stimulation (Fig. 5). Based solely on these experiments we cannot dismiss the hypothesis by Gangloff [16], even though we did observe some TRIF-dependent activation. Since there are some contradictory reports on whether the MyD88-independent pathway is truly triggered solely from the acidic endosome vesicles, we could consider the possibility that some activation of the TRIF-dependent pathway occurs either from the plasma membrane or from the surface of early endosomes that have not yet been acidified. Moreover, an interesting recent study by Zoroddu et al. [29] has shown that acidic environment may not necessarily interfere with nickel coordination by histidine residues. By using a peptide fragment of the hTLR4 ectodomain that encompassed the histidine residues of interest, they have shown that nickel could bind the histidines at both physiological as well as low pH [29]. These results indicate that even if TRIF-dependent pathway can be triggered exclusively from the acidic endosome vesicles, that does not prevent its activation by nickel.

**Fig 6:**
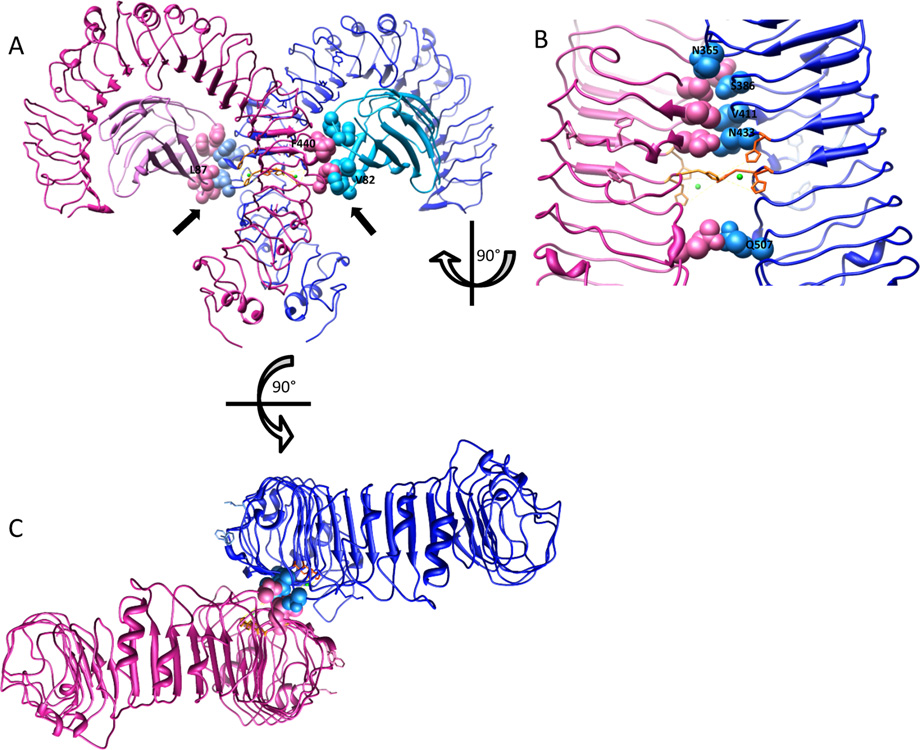
MD-2 provides crucial stabilization that supports the formation of TLR4/MD-2 heterodimer with nickel or cobalt ions. (A) A ribbon representation of a TLR4/MD-2 heterodimer (pdb id 3fxi). Amino acid residues of both TLR4 and MD-2 that form hydrophobic interactions with each other are shown as spheres and indicated with arrows. (B) A ribbon representation of the intrinsic dimerization interface between both TLR4 ectodomains. Amino acid residues that engage in direct interactions with one another are shown as spheres. Histidines H431, H456 and H458 are represented as sticks and colored orange. Green spheres represent metal ions that are coordinated between the indicated histidines. (C) A ribbon representation of TLR4 dimer without MD-2 shown from a top view. Without MD-2 both TLR4 ectodomains lack proper stabilization and can wobble around one another, causing disruption of a proper conformation that would enable cytoplasmic TIR domain dimerization and consecutive triggering of the TLR4 receptor signaling pathway. Figures were prepared with the UCSF Chimera package [31].

Based on our experiments and observations of others [10,11] we propose a model of TLR4/MD-2 activation by transition metal ions (Fig. 6), where MD-2 provides supporting hydrophobic interactions with TLR4, which together with the intrinsic interactions between the TLR4 ectodomains [6] on top of the metal ion coordination interactions, cumulates to the dimerization of TLR4 receptor ectodomains and their respective cytoplasmic TIR-domains that leads to cell activation. Without hydrophobic interactions between MD-2 and TLR4 the metal-bound TLR4 ectodomain dimer lacks proper stabilization, causing disruption of a proper conformation that would enable TIR domain dimerization and consecutive triggering of the TLR4 receptor signaling pathway (Fig. 6). Contribution of all interactions in an activated heterodimer (i.e. hydrophobic interactions between MD-2 and TLR4, intrinsic interactions between both TLR4 ectodomains and the metal-induced interactions mediated via the histidine residues of the TLR4 ectodomains) is necessary to enable strong cell activation. This is underlined by a recent study, which provided evidence that even a small disturbance in these interactions can prevent TLR4 dimerization; a TLR4 ectodomain with a mutation in the residue N433 that is involved in intrinsic interactions between the TLR4 ectodomains, was unable to co-immunoprecipitate in the presence of nickel or cobalt ions [11]. Surprisingly, an isolated TLR4 ectodomain can apparently dimerize with the help of nickel or cobalt *in vitro* without MD-2, but no evidence was provided to show whether this dimer would be stable enough and in a proper conformation to support receptor activation. An early study reported that TLR4 cannot reach the plasma membrane without the co-expression of MD-2, which could explain the requirement for MD-2 to be present *in vivo* [30]. This was shown for a single cell type and latter studies have provided evidence of TLR4 expression on the plasma membrane without the help of MD-2. Therefore our results lead us to infer that without the stabilization provided by MD-2, the interactions between TLR4 ectodomains in the dimer are too weak and unstable to enable cell activation.

This work offers further insight into the role of TLR4 in the innate response to transition metals in humans and provides an explanation for the need for the presence of MD-2 in the activating complex despite not being directly involved in metal ion binding.
